# Association of serum zinc level and clinical outcome in Egyptian COVID-19 patients

**DOI:** 10.1186/s43162-022-00159-z

**Published:** 2022-09-24

**Authors:** Ahmed M. F. Mansour, Noha El Nakeeb, Norhan Khaled Mohamed Kamal, Ahmed Mohamed ElGhandour

**Affiliations:** Department of Internal Medicine, Hepatology and Gastroenterology, Ain Shams University, Cairo, Egypt

**Keywords:** COVID-19, Zinc, Zn, Immunity

## Abstract

**Background:**

Zinc is an anti-inflammatory and antioxidant micronutrient found in food. Due to its well-established role in immunity, it is currently being used in some clinical trials against coronavirus disease-2019 (COVID-19). This study aimed to assess the association between the mean serum zinc level in COVID-19 Egyptian patients and its relationship with disease severity. This cross-sectional study was conducted on sixty patients with confirmed COVID-19 infection. These patients were divided into two groups according to clinical outcome, group 1 which included 30 intensive care unit (ICU) patients and group 2 which included 30 patients who were admitted to the ward. Mean serum levels of zinc were compared between the two groups.

**Results:**

There was a statistically significant difference noted among study groups regarding the serum zinc level (*p* < 0.039), where lower mean serum zinc levels were noted in ICU patients compared to ward patients (70.6 ± 5.7 vs 73.8 ± 6.1).

**Conclusion:**

Low serum zinc level is associated with the severe outcome of COVID-19 infection.

## Background

Through the last 2 years, the world was devastated by a serious pandemic of COVID-19 caused by the severe acute respiratory syndrome coronavirus-2 (SARS-CoV-2). Since then, researchers from all over the world carefully studied any possible aetiologies or risk factors to help in finding effective treatment regimens based on current knowledge that can help control the spread of the disease with the help of currently available vaccines in the hope to prevent further pandemic flares [1].

Therapeutic approaches against COVID-19 are currently focusing on the management of its immunopathology and/or tailored to directly control viral replication. Several human trials are currently in progress to assess the therapeutic indices of different drugs, and in combination with dietary supplements like vitamin C, vitamin D, vitamin B12, probiotics, and zinc [1].

Zinc is the second most common trace element that is vital for the growth, development, and maintenance of immune function, in addition to its critical role in antiviral immunity. Its influence reaches all organs and cell types, representing an essential component of approximately 10% of the human proteome and encompassing hundreds of key enzymes and transcription factors [2, 3]. Many foods are considered rich in zinc including meat, poultry, shellfish, legumes, nuts, eggs, and dairy products [4]. The recommended daily intake of zinc for adult males and females is 11 mg and 8 mg, respectively. Pregnant and breastfeeding females need higher daily intake reaching 11 mg and 12 mg, respectively [5].

Zinc deficiency is common. The global prevalence of zinc deficiency is estimated to range from 17 to 20% [6, 7], with the vast majority occurring in developing countries of Africa and Asia. High-risk factors for zinc deficiency include patients with gastrointestinal diseases such as Crohn’s disease; patients with sickle cell anemia, malnutrition, or chronic kidney disease; and vegetarians and vegans, in addition to pregnant and breastfeeding females [7].

Zinc is a common theme in both prophylactic and curative COVID-19 clinical studies using nutritional supplements. Thus, this study aimed to assess the possible association between mean levels of serum Zn and clinical outcomes in Egyptian COVID-19 patients.

## Methods

This comparative cross-sectional study was conducted on 60 Egyptian COVID-19 patients (35 males (58.3%) and 25 females (41.7%), mean age: 59.2 ± 15.3 years) recruited from our institution’s quarantine field hospital and quarantine university hospital ICU and inpatient wards during the period from July to December 2021. These patients were divided into two groups according to the severity of COVID-19 infection; the ICU group included 30 patients with severe COVID-19 infection who were admitted to ICU, while the Word group included 30 patients with moderate COVID-19 infection admitted to inpatient wards.

All patients were diagnosed with COVID-19 infection by polymerase chain reaction (PCR) of oropharyngeal and nasopharyngeal swabs. Laboratory investigations were carried out for all recruited patients and included complete blood count, erythrocyte sedimentation rate (ESR), C-reactive protein (CRP), serum creatinine, alanine aminotransferase (ALT), aspartate aminotransferase (AST), serum albumin, serum ferritin, lactate dehydrogenase (LDH), and D-dimer, in addition to serum zinc level. All patients underwent high-resolution computed tomography (HRCT) of the chest to assess the severity of lung affection due to COVID-19 infection. All patients were receiving zinc supplements in the form of zinc sulfate heptahydrate equivalent to 75–100 mg elemental zinc as per COVID-19 national guidelines. All collected data was tabulated, interpreted, and statistically analyzed.

## Results

Patients included in this study were 21 smokers (35%) and 39 non-smokers (65%). Other co-morbidities were noted, where 43 patients (71.7%) had co-morbidities such as hypertension, diabetes mellitus, or both, in addition to atrial fibrillation (AF). Seventeen patients (28.3%) had no co-morbidities.

Comparison between both groups as regards demographic data showed that there was no statistically significant difference found between the two groups regarding gender, age, and smoking.

Comparison between both groups as regards co-morbidities revealed a statistically significant difference (*p* < 0.005). Moreover, it was noted that patients with combined diabetes and hypertension (*n* = 15) had a more severe course of infection demanding admission to ICU (*n* = 11, 73.3%) rather than the inpatient ward (*n* = 4, 26.7%). This was also similar to patients with both hypertension and AF, where they were all admitted to ICU (*n* = 3, 100%). This can elucidate the possible risk multiple co-morbidities convey on the course of infection in COVID-19 patients (Table 1).

**Table 1:** Comparison between ward and ICU patients regarding CO-RADS classification

		**Admission**						**Chi-square**	
		**Ward**		**ICU**		**Total**			
		***N***	**%**	***N***	**%**	***N***	**%**	***X***^**2**^	***p***
**Medical history**	**Free**	11	36.67	6	20.00	17	28.33	14.959	0.005
	**DM**	7	23.33	0	0.00	7	11.67		
	**HTN**	8	26.67	10	33.33	18	30.00		
	**DM + HTN**	4	13.33	11	36.67	15	25.00		
	**HTN + AF**	0	0.00	3	10.00	3	5.00		
**CO-RADS** **classification**	**CO-RADS 3**	19	63.33	9	30.00	28	46.67	7.413	0.025
	**CO-RADS 4**	6	20.00	8	26.67	14	23.33		
	**CO-RADS 5**	5	16.67	13	43.33	18	30.00		

*p* > 0.05: non-significant (NS); *p* < 0.05: significant (S); *p* < 0.001: highly significant (HS)

Similarly, a comparison between study groups as regards CO-RADS classification revealed a statistically significant difference (*p* < 0.025). It was also noted that the risk for admission to ICU increased as CO-RADS grade increased. This goes with the fact that a higher CO-RADS grade indicated more severe infection, which necessitated more intensive management in critical care settings. Patients with CO-RADS grade 5 had a much higher risk of being admitted to ICU (*n* = 13, 72.2%) rather than the inpatient ward (*n* = 5, 27.8%) (Table 1).

A comparison of different laboratory parameters of both study groups showed a significant statistical difference among patients as regards total leucocytic count (TLC), neutrophil count, neutrophil/lymphocyte ratio, CRP, and ESR (*p* < 0.005, *p* < 0.004, *p* < 0.015, *p* < 0.048, and *p* < 0.029, respectively). Moreover, there was a statistically significant difference between study groups as regards ferritin, LDH, and D-dimer (*p* < 0.013, *p* < 0.042, and *p* < 0.002, respectively), indicating a more severe course of COVID-19 in ICU patients. There was also a significant statistical difference between study groups as regards AST and ALT (*p* < 0.052 and *p* < 0.027, respectively), in addition to a highly significant statistical difference among study groups as regards vitamin D levels (*p* < 0.001), where a significant decline in mean vitamin D levels was noticed in ICU patients compared to ward patients (18.3 ± 4.8 vs 23.1 ± 3.9) (Table 2).

**Table 2:** Comparison between study groups as regards different laboratory parameters

		**Admission**						***t*-test**	
		**Ward**			**ICU**			***t***	***p***
**TLC**	**Range**	2.3	–	20.8	3.3	–	33	− 2.922	0.005
	**Mean ± SD**	7.650	±	4.491	12.520	±	7.948		
**Lymphocytes**	**Range**	0.3	–	2	0.2	–	1.7	0.822	0.415
	**Mean ± SD**	0.750	±	0.432	0.657	±	0.448		
**Neutrophils**	**Range**	1	–	18	2.2	–	31.9	− 2.981	0.004
	**Mean ± SD**	6.303	±	4.261	11.050	±	7.610		
**Hemoglobin**	**Range**	6.5	–	18.5	7	–	18	0.334	0.739
	**Mean ± SD**	12.320	±	2.621	12.110	±	2.229		
**Platelets**	**Range**	17	–	515	112	–	398	− 0.412	0.682
	**Mean ± SD**	200.433	±	91.409	209.267	±	73.555		
**Neutrophil-** **lymphocyte ratio**	**Range**	1	–	43	2	–	106	− 2.498	0.015
	**Mean ± SD**	12.967	±	13.330	25.567	±	24.196		
**CRP**	**Range**	0.1	–	300	4.5	–	330	− 2.019	0.048
	**Mean ± SD**	47.049	±	70.470	92.331	±	100.650		
**ESR**	**Range**	5.1	–	120	2	–	300	− 2.242	0.029
	**Mean ± SD**	30.617	±	32.174	62.190	±	70.114		
**Creatinine**	**Range**	0.3	–	9	0.4	–	6.1	− 0.607	0.546
	**Mean ± SD**	1.273	±	1.594	1.490	±	1.132		
**AST**	**Range**	9	–	215	4	–	212	− 1.988	0.052
	**Mean ± SD**	38.067	±	41.047	61.700	±	50.545		
**ALT**	**Range**	9	–	185	13	–	338	− 2.272	0.027
	**Mean ± SD**	37.033	±	35.762	76.500	±	88.160		
**Albumin**	**Range**	1.8	–	3.9	2.1	–	4.2	1.556	0.125
	**Mean ± SD**	3.327	±	0.470	3.123	±	0.540		
**Ferritin**	**Range**	56	–	1255	26.7	–	2100	− 2.569	0.013
	**Mean ± SD**	369.773	±	302.147	642.770	±	497.362		
**LDH**	**Range**	110	–	963	125	–	986	− 2.081	0.042
	**Mean ± SD**	379.667	±	256.082	518.967	±	262.309		
**D-dimer**	**Range**	0.2	–	2.9	0.212	–	6.9	− 3.303	0.002
	**Mean ± SD**	0.737	±	0.715	1.806	±	1.621		
**Vitamin D**	**Range**	15	–	30	7	–	27	4.310	< 0.001
	**Mean ± SD**	23.133	±	3.902	18.267	±	4.799		

*p* > 0.05: non-significant (NS); *p* < 0.05: significant (S); *p* < 0.001: highly significant (HS)

As regards serum zinc levels, there was a statistically significant difference noted among study groups (*p* < 0.039), where lower mean serum zinc levels were noted in ICU patients compared to ward patients (70.6 ± 5.7 vs 73.8 ± 6.1). These results further elucidate the potential protective role of serum zinc due to its significant role in the maintenance of host’s immune functions in addition to its potential antiviral effects. Moreover, observations from this study point towards the effects of zinc deficiency on the natural course of COVID-19 infection and the possibilities of poor prognosis in patients due to severe infection (Table 3).

**Table 3:** Comparison between ward and ICU regarding serum zinc level

**Zinc level**	**Admission**						***t*-test**	
	**Ward**			**ICU**			***t***	***p***
**Range**	65	–	89	56	–	81	2.114	0.039
**Mean ± SD**	73.800	±	6.059	70.600	±	5.661		

*p* > 0.05: non-significant (NS); *p* < 0.05: significant (S); *p* < 0.001: highly significant (HS)

## Discussion

SARS-CoV is a virus which is capable to bind the angiotensin-converting enzyme 2 receptors that are present in several locations such as lung alveolar epithelial cells, enterocytes, endothelial cells, and arterial smooth muscle cells in the human body. This novel coronavirus has caused many deaths in the recent pandemic. Evidence suggested that in severe COVID-19 cases, a cytokine storm accompanies the infection [8]*.* Several studies focused on possible risk factors that may contribute to immunopathogenesis or the natural course of infection in COVID-19 patients. The present study aimed to assess the association between mean serum levels of zinc and the severity of infection in Egyptian COVID-19 patients.

Earlier studies demonstrated that a decreased zinc level favors the interaction of ACE2 with SARS-CoV-2 spike protein and likewise that an increased zinc level inhibits ACE2 expression resulting in reduced viral interaction [9, 10]*.* On the other hand, the first clinical study correlating lower baseline zinc levels in patients with COVID-19 was done by Jothimani et al., and it was compared to healthy controls showing a highly significant statistical difference between the two groups as regards mean serum zinc levels (74.5 vs 105.8 μg/dL, *p* < 0.001). Among COVID-19 patients in that study, 57.4% (*n* = 27) were zinc deficient [11].

Moreover, Shakoor and his colleagues recently discussed zinc’s role in immunity and its effect on patients with COVID-19. They also discussed consuming this nutrient as a potential therapeutic method for reducing the complications and mortality rate of patients with COVID-19 [12]. This agreed with results from another study by Foster et al., where they proved that zinc supplements positively affected reducing fever duration in patients with respiratory infections. Still, it had no significant effect on respiratory rate, cough duration, and hospitalization time [13].

Based on Abdolahi et al. study findings, zinc’s serum level had a significant difference between 93 hospitalized COVID-19 patients compared to 186 healthy subjects, where the case group had a lower serum zinc level than the control group, and the study suggested that zinc deficiency could be a predictor for a critical illness of COVID-19. Moreover, the study recommended using zinc supplements for non-patients for prevention and for patients who may have lower than normal serum zinc levels. However, trials with an increased number of patients should still be evaluated [14].

Several surveys conducted have claimed that zinc consumption is likely to reduce the intensity of COVID-19 infection due to its antiviral properties; it also helps to alleviate respiratory tract infections [3, 15].

There are few published studies that illustrate the efficacy of zinc therapy in managing COVID-19 patients [16–18]. Many individuals globally consume zinc tablets and vitamins C and B due to possible immune booster effects for protection against COVID-19 infection [19, 20].

However, a recent retrospective analysis utilizing electronic medical records found that patients treated with hydroxychloroquine and azithromycin with the addition of zinc sulfate had a higher recovery rate. Interestingly, additional input of zinc sulfate was claimed to be associated with lower mortality rate, need for hospice care, and less invasive ventilation requirements. However, this association was not observed among ICU patients [21].

González and his colleagues demonstrated in their study a correlation between serum zinc levels and COVID-19 outcome. Serum zinc levels lower than < 50 μg/dL at admission correlated with worse clinical presentation, longer time to recovery, and higher mortality. These results, in addition to several other recent studies, might suggest that serum zinc impacts COVID-19 severity, and its adjustment might also constitute an early therapeutic intervention point [22–24]. Another in vitro study by Velthuis et al. showed that Zn^+2^ cations especially in combination with zinc ionophore pyrithione were shown to inhibit SARS-coronavirus RNA polymerase activity by decreasing its replication [25].

## Conclusion

Low serum zinc level is associated with severe COVID-19 infection. This is likely due to a combination of immune system imbalance and a direct benefit of viral replication. Thus, we propose serum zinc level as a novel and additional parameter to predict COVID-19 outcomes. Moreover, this study recommends implementing nutritional education programs for healthcare professionals, nursing staff, and dieticians to help raise public awareness concerning healthy foods and eating habits, including zinc-rich food sources. It is also recommended to implement good physician-pharmacist teamwork when using food supplements to maximize benefits and avoid potential drug-drug interactions.

## Data Availability

The datasets used and/or analyzed during the current study are available from the corresponding author on reasonable request.

## Abbreviations

AF: Atrial fibrillation
ALT: Alanine aminotransferase
AST: Aspartate aminotransferase
COVID-19: Coronavirus disease-2019
CRP: C-reactive protein
ESR: Erythrocyte sedimentation rate
HRCT: High-resolution computed tomography
ICU: Intensive care unit
LDH: Lactate dehydrogenase
PCR: Polymerase chain reaction
SARS-CoV-2: Severe acute respiratory syndrome coronavirus-2
